# Inflammasome-triggered IL-18 controls skin inflammation in the progression of Buruli ulcer

**DOI:** 10.1371/journal.ppat.1011747

**Published:** 2023-11-01

**Authors:** Toshihiko Suzuki, Kotchakorn Boonyaleka, Tokuju Okano, Tamako Iida, Mitsunori Yoshida, Hanako Fukano, Yoshihiko Hoshino, Yoichiro Iwakura, Anthony S. Ablordey, Hiroshi Ashida

**Affiliations:** 1 Department of Bacterial Pathogenesis, Infection and Host Response, Graduate School of Medical and Dental Sciences, Tokyo Medical and Dental University (TMDU), Tokyo, Japan; 2 Department of Mycobacteriology, Leprosy Research Center, National Institute of Infectious Diseases, Tokyo, Japan; 3 Research Institute for Biomedical Sciences, Tokyo University of Science, Noda, Chiba, Japan; 4 Department of Bacteriology, Noguchi Memorial Institute for Medical Research, University of Ghana, Accra, Ghana; UW, UNITED STATES

## Abstract

Buruli ulcer is an emerging chronic infectious skin disease caused by *Mycobacterium ulcerans*. Mycolactone, an exotoxin produced by the bacterium, is the only identified virulence factor so far, but the functions of this toxin and the mechanisms of disease progression remain unclear. By interfering Sec61 translocon, mycolactone inhibits the Sec61-dependent co-translational translocation of newly synthesized proteins, such as induced cytokines and immune cell receptors, into the endoplasmic reticulum. However, in regard to IL-1β, which is secreted by a Sec61-independent mechanism, mycolactone has been shown to induce IL-1β secretion via activation of inflammasomes. In this study, we clarified that cytokine induction, including that of IL-1β, in infected macrophages was suppressed by mycolactone produced by *M*. *ulcerans* subsp. *shinshuense*, despite the activation of caspase-1 through the inflammasome activation triggered in a manner independent of mycolactone. Intriguingly, mycolactone suppressed the expression of proIL-1β as well as TNF-α at the transcriptional level, suggesting that mycolactone of *M*. *ulcerans* subsp. *shinshuense* may exert additional inhibitory effect on proIL-1β expression. Remarkably, constitutively produced IL-18 was cleaved and mature IL-18 was actually released from macrophages infected with the causative mycobacterium. IL-18-deficient mice infected subcutaneously with *M*. *ulcerans* exhibited exacerbated skin inflammation during the course of disease progression. On the other hand, IL-1β controls bacterial multiplication in skin tissues. These results provide information regarding the mechanisms and functions of the induced cytokines in the pathology of Buruli ulcer.

## Author summary

Buruli ulcer, caused by *Mycobacterium ulcerans*, is an emerging chronic disease of the skin, characterized by massive skin ulceration. Mycolactone, an exotoxin produced by *M*. *ulcerans* is the sole identified virulence factor, but its functions are still unclear. By interfering Sec61 translocon, mycolactone inhibits Sec61-dependent co-translational translocation of newly synthesized proteins, such as induced cytokines and immune cell receptors, into the endoplasmic reticulum. However, in regard to IL-1β, which is secreted by a Sec61-independent mechanism, mycolactone has been shown to induce IL-1β secretion. In this study, we analyzed the activation of inflammasomes in infected macrophages using wild-type and toxin-negative strains of *M*. *ulcerans* subsp. *shinshuense*, and found that mycolactone inhibited IL-1β secretion, but not IL-18, resulting in the exclusive induction of IL-18 by the bacterial infection. Intriguingly, mycolactone suppressed the expression of proIL-1β as well as TNF-α at the transcriptional level, suggesting that mycolactone of *M*. *ulcerans* subsp. *shinshuense* may exert additional inhibitory effect on proIL-1β expression. Inflammasome activation accompanied by caspase-1 activation in cases of Buruli ulcer appears to be triggered in a manner largely independent of mycolactone. Using a mouse model of Buruli ulcer, we further demonstrated that IL-18 attenuates the progression of Buruli ulcer by controlling accumulation of neutrophils in the infected skin lesions. On the other hand, IL-1β controls bacterial multiplication in the skin. Our results shed light on the etiology of Buruli ulcer and provide insight into the relationship between the activation of inflammasomes and functions of cytokines.

## Introduction

Buruli ulcer (BU) is a chronic skin disease characterized by massive skin ulceration. It is an emerging infectious disease caused by *Mycobacterium ulcerans*, and has been defined as a neglected tropical disease by the World Health Organization (WHO). BU has been reported in more than 30 countries worldwide, with areas of endemicity emerging in Africa and Australia [1,2]. Absence of pain is a major characteristic of the skin lesions in BU, and the absence of other overt signs of infection in the disease often delays the diagnosis. If left untreated, the necrotic skin ulcers and soft tissue destruction could extend up to 15% of the body surface area, causing both disfigurement and disability [3]. Treatment with antibiotics may be effective in the early phase of disease, but surgical procedures such as debridement and skin grafting may be necessary in more severe cases, or even limb amputation in extreme cases [4].

The typical host immune response to mycobacterial infections such as *M*. *tuberculosis* or *M*. *leprae* is the formation of the characteristic granuloma, an organized structure composed of immune cells surrounding the infecting mycobacteria. In contrast, BU lesions are characterized by clusters of extracellular bacilli within necrotic tissue, with a relative paucity of infiltrating leukocytes [5,6]. These pathological features are due to the exotoxin called mycolactone, a unique virulence factor secreted by *M*. *ulcerans* [7].

Mycolactone, produced by *M*. *ulcerans*, is encoded by a large plasmid and plays a central role in the pathogenesis of BU [8]. Mycolactone suppresses both innate and adaptive immune responses in the hosts, by abrogating the production of cytokines and chemokines by immune cells such as macrophages and T cells, and also preventing induction immune receptors on the cell surface and antigen presentation [6,9–15].

These biological actions of mycolactone are exerted via toxin-mediated interference to Sec61 translocon, which results in inhibition of Sec61-dependent co-translational translocation of newly synthesized proteins into the endoplasmic reticulum (ER) [12]. In turn, this results in inhibition of secretory proteins, such as cytokines and immune receptors induced by bacterial infection, suggesting that the toxin may confer immune evasion properties [14,16]. Also, by targeting AT2R receptors, the toxin is thought to be contribute to the absence of pain in the skin ulcers of BU [17,18]. Furthermore, the toxin also exerts cytotoxic activity, inducing tissue destruction and necrosis through a mechanism that might involve its interaction with Sec61 [7,19,20].

Although the biological activities of mycolactone during infection are thought to mediate immunosuppressive effects, recent studies have shown that exposure to a low dose of mycolactone can trigger NLRP3 inflammasome activation resulting in IL-1β release from LPS-primed human macrophages [21,22]. Infection with *M*. *ulcerans* and treatment with extracellular vesicles containing mycolactone revealed that the production of IL-1β by human macrophages occurred in a mycolactone-dependent manner [21]. The secretion of IL-1β as well as that of IL-18 is regulated by the inflammasome. Activation of inflammasomes results in conversion of caspase-1 to its active form, which, in turn, proteolytically processes proIL-1β and proIL-18 to produce active cytokines. The family of Nod-like receptor (NLR) finely regulates caspase-1 activation in response to extracellular stimuli [23]. The secretion pathway of these cytokines is mechanistically distinctive as compared with that for other cytokines such as TNF-α. Recent study showed that mycolactone stimulates IL-1β secretion by activating the NLRP3 inflammasome [21]. On the other hand, mycolactone inhibits production of IL-1β, as well as that of other cytokines that are induced by TLR ligands [11]. Thus, to date, the precise molecular mechanisms underlying mycolactone-mediated inflammasome activation and its role in disease progression remain controversial. How inflammasome activation and consequent induction of inflammatory cytokines contributes to the progression of BU also remains unclear.

In the present study, we used an in vitro infection model using both viable wild-type *M*. *ulcerans* subsp. *shinshuense*, and mycolactone-deficient plasmid-cured strain to investigate the induction of cytokines and activation of inflammasomes in infected murine macrophages. The secretion of IL-1β was inhibited in a mycolactone-dependent manner, despite the activation of caspase-1 via inflammasome activation. Unexpectedly, mycolactone also suppressed the proIL-1β expression at the transcriptional level through a Sec61-independent mechanism. On the other hand, inflammasome activation was triggered in a manner that was largely independent of mycolactone. Remarkably, constitutively produced proIL-18 was cleaved and mature IL-18 was actually released from the macrophages infected with wild-type *M*. *ulcerans*. We also examined the functional roles of IL-18 in disease progression using an IL-18-deficient mouse model with subcutaneous infection. We found that IL-18 negatively regulated the exacerbation of skin inflammation induced by the bacterial infection, whereas IL-1β was involved in the control of the bacterial burden.

Taken together, the above results suggest that IL-18, which is induced in a mycolactone-independent manner, plays a key role in the regulation of inflammation.

## Results

### *M*. *ulcerans* exclusively induces IL-18 secretion through inflammasome activation

**Fig 1:**
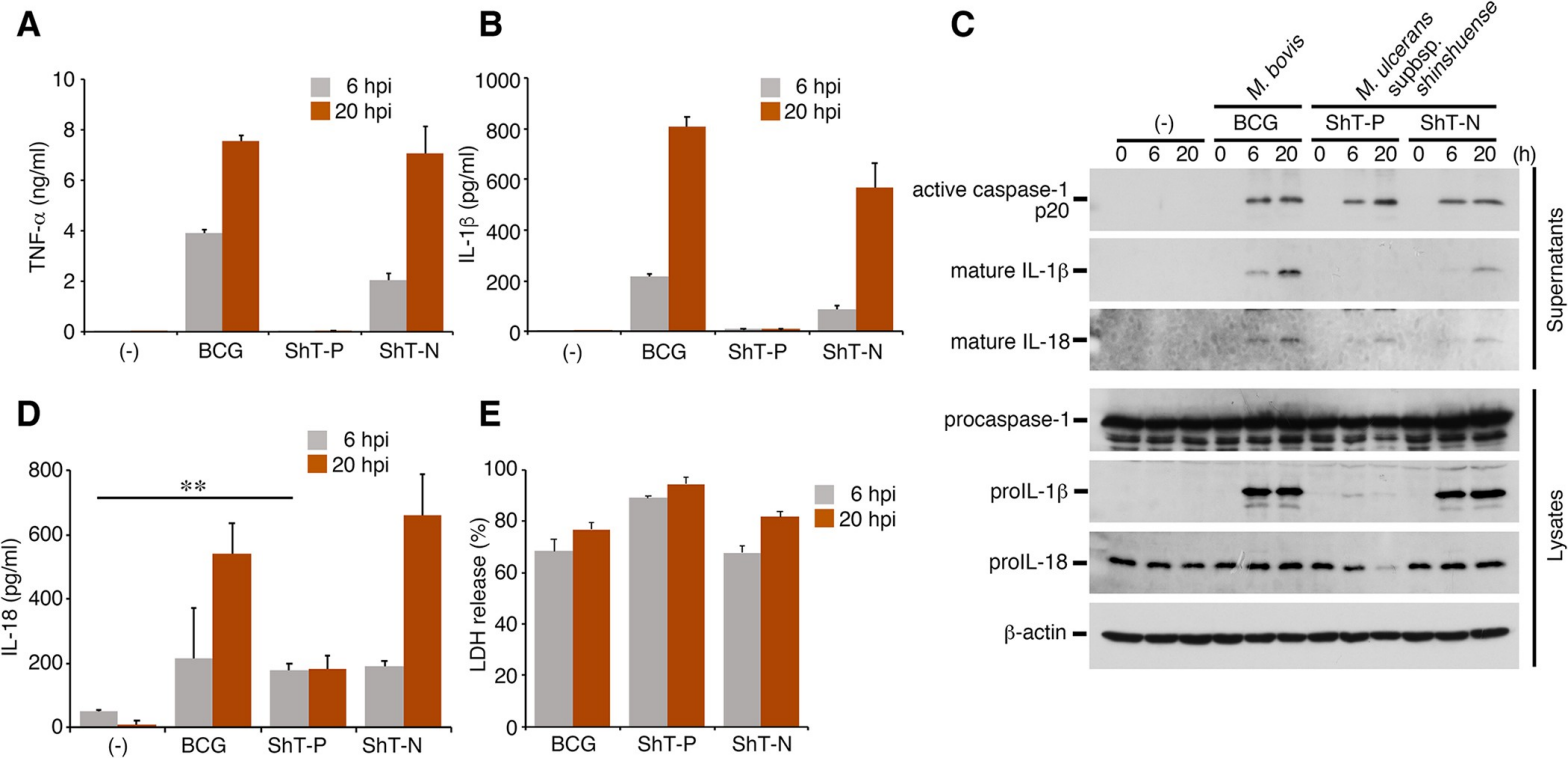
*M*. *ulcerans* exclusively induces IL-18 secretion through inflammasome activation. (A) TNF-α production, (B) IL-1β production, (D) IL-18 production, as determined by ELISA, in the supernatants of bone marrow-derived macrophages (BMDMs) obtained from WT mice at 6 or 20 h after infection with the *M*. *ulcerans* strains. (C) Results of Western blot analysis showing representative cleavages and secretions of caspase-1, IL-1β, and IL-18 at 20 h after infection. (E) LDH release from the infected macrophages; representative of at least 3 experiments. The error bars represent the means ± SD. **P < 0.01; two-tailed unpaired *t* tests for (D).

The inflammatory responses of resident macrophages in the skin play a major role in protecting the skin tissue against infection with pathogenic microbes. To investigate the inflammatory responses induced by *M*. *ulcerans* infection, we used the *M*. *ulcerans* subsp. *shinshuense*, a causative agent of BU in Japan, to infect mouse bone marrow-derived macrophages (BMDMs) and analyze the subsequent induction of cytokines. This wild-type strain ShT-P produces mycolactone A/B, S1 and S2 [24]. Consistent with previous reports [12,15], TNF-α was not induced upon infection with wild-type *M*. *ulcerans* ShT-P strain (Fig 1A). Furthermore, we found that IL-1β was also not induced after infection with wild-type *M*. *ulcerans* (Fig 1B). In contrast, macrophages infected with the *M*. *bovis* BCG strain or the plasmid-cured *M*. *ulcerans* ShT-N strain (which does not produce mycolactone) secreted remarkable amounts of TNF-α and IL-1β (Fig 1A and 1B). Western blot analysis revealed that the levels of cytosolic proIL-1β and cleaved IL-1β (the biologically active mature form) in the supernatants were markedly reduced following infection with the wild-type strain, but not after infection with the BCG or ShT-N strain (Fig 1C). These data suggest that mycolactone suppresses induction of the IL-1β protein, resulting in markedly reduced secretion of IL-1β as well as TNF-α from the infected macrophages.

The mature form of IL-1β is generated by caspase-1, which is activated by a cytosolic multiprotein platform known as an inflammasome. We examined caspase-1 activation in macrophages infected with the wild-type *M*. *ulcerans* and plasmid-cured *M*. *ulcerans* strains. Intriguingly, caspase-1 was activated upon infection with wild-type *M*. *ulcerans* as well as infection with a plasmid-cured strain, suggesting that capase-1 activation is induced in a mycolactone-independent manner. We then focused on another inflammatory cytokine, IL-18; this cytokine is also activated by caspase-1 as well as IL-1β. Mycolactone exerted little effect on constitutively produced proIL-18; furthermore, significant amounts of mature IL-18 were secreted into the supernatant of cells infected with wild-type *M*. *ulcerans* (Fig 1C and 1D). No difference in lactate dehydrogenase (LDH) release induced by caspase-1 activation was seen between cells infected with the wild-type and plasmid-cured strains of *M*. *ulcerans* (Fig 1E). These results suggest that infection with wild-type *M*. *ulcerans* exclusively induces IL-18 secretion from the infected macrophages.

**Fig 2:**
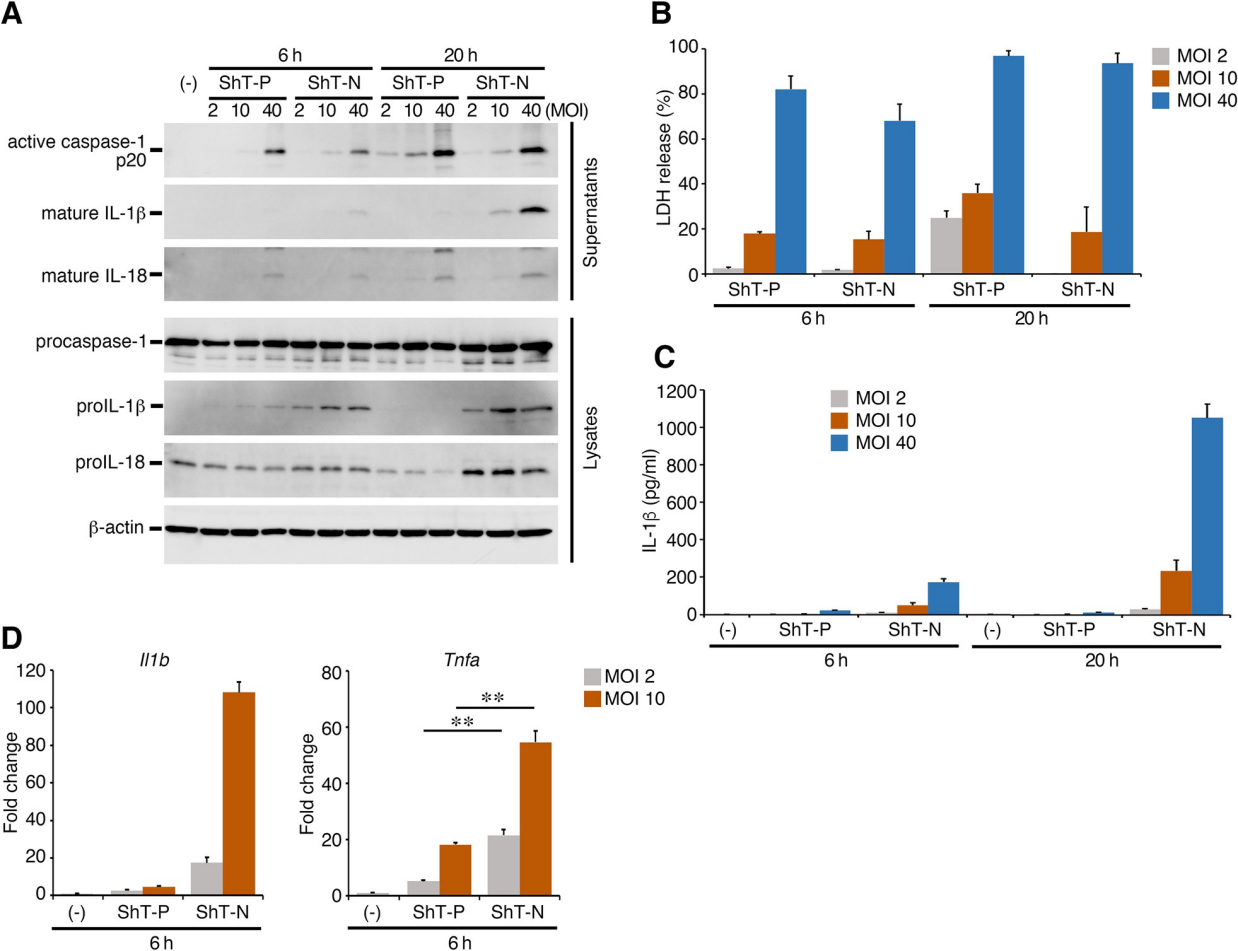
Mycolactone, an exotoxin produced by *M*. *ulcerans*, suppresses proIL-1β expression even after infection at a low MOI. (A) Results of Western blot analysis showing representative cleavages and secretions of caspase-1, IL-1β, and IL-18 after infection at a MOI of 2, 10 or 40. (B) LDH release from the infected macrophages. (C) IL-1β production as determined by ELISA. (D) RT-qPCR analysis to determine the transcription levels of IL-1β and TNF-α at a MOI of 2 or 10 for 6 h. Relative fold change in gene expression was calculated using uninfected cells as the control; representative of at least 3 experiments. The error bars represent the means ± SD. **P < 0.01; one-way ANOVA for (D).

To clarify the unexpected inhibitory effect of mycolactone observed on proIL-1β expression, we examined the inflammatory responses induced in macrophages infected at a low multiplicity of infection (MOI). The expression of proIL-1β was still suppressed by infection with the wild-type ShT-P strain as compared with the ShT-N at an MOI of 2 or 10 (Fig 2A). Infection at an MOI of 2 or 10 also did not induce high value of LDH release (Fig 2B), suggesting that the suppression of proIL-1β expression by infection with the wild-type *M*. *ulcerans* was not a result of cell death of the infected macrophages. On the other hand, caspase-1 was activated on MOI-dependent manner and closely correlated with the amounts of LDH release (Figs 1B and 2A). The activation was slightly enhanced in the macrophages infected with the mycolactone-expressing wild-type strain at a low MOI (band density of cleaved caspase-1 at 20 h at a MOI of 2) as compared with the macrophages infected with ShT-N, suggesting partial contribution of mycolactone to inflammasome activation. The amounts of IL-1β released reflected the expression level of proIL-1β and state of activation of caspase-1 (Fig 2C). Next, we examined the transcription level of proIL-1β and TNF-α in the macrophages at an MOI of 2 or 10 for 6 h after infection with *M*. *ulcerans*. As shown in Fig 2D, mRNA expression of proIL-1β was markedly suppressed in the infection with wild-type ShT-P strain, suggesting that mycolactone inhibits the proIL-1β expression at the transcriptional level. Mycolactone suppresses the induced cytokines such as TNF-α by interfering Sec61 translocon. However, not as conspicuous as the inhibition of proIL-1β mRNA, the toxin significantly inhibited TNF-α mRNA induction as compared with toxin-negative ShT-N infection. These data suggest the possibility that mycolactone inhibits the induction of induced cytokines at the transcriptional level by a mechanism independent of the Sec61-mediated protein secretion system.

The induction of inflammatory cytokines was also examined in macrophages infected with another *M*. *ulcerans* strain, Agy99, or related strains *M*. *marinum* or *M*. *pseudoshottsii* (S1 Fig). The mycolactone A/B-producing Agy99 strain [25] apparently did not suppress proIL-1β expression, but rather induced the secretion of small amounts of IL-1β and TNF-α (S1C Fig). The level of caspase-1 activation was comparatively lower, resulting in a low level of IL-1β secretion. The infected cells also released a low level of LDH (S1B and S1C Fig). These results suggest that the level of suppression of cytokines and caspase-1 activation might differ depending on the infecting strain of *M*. *ulcerans*. Since the *M*. *marinum* strain does not produce mycolactone, large amounts of cytokines were released from the cells infected with this strain (S1A and S1B Fig). On the other hand, similar to the *M*. *ulcerans* subsp. *shinshuense*, infection with mycolactone F-producing *M*. *pseudoshottsii* [26] exclusively induced IL-18 production (S1D Fig), suggesting that the mycolactone-mediated suppression of cytokines is not an *M*. *ulcerans*-specific phenomenon.

### *M*. *ulcerans* triggers inflammasome activation in a manner dependent on ASC but not on NLRP3, NLRP6, AIM2 or non-canonical pathways induced by caspase-11

**Fig 3:**
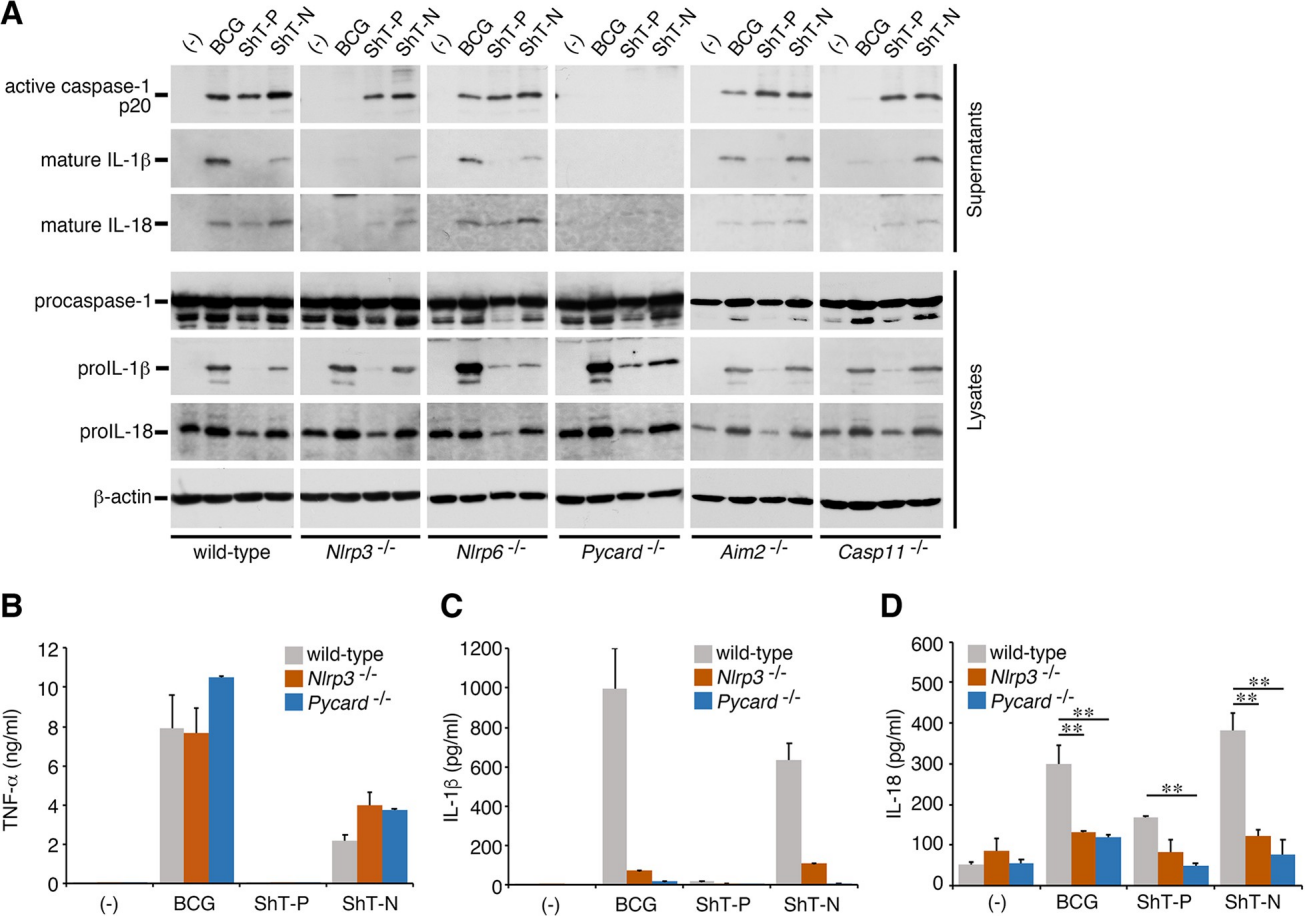
*M*. *ulcerans* triggers inflammasome activation in a manner dependent on ASC but not on NLRP3, NLRP6, AIM2 or non-canonical pathways inducted by caspase-11. (A) Results of Western blot analysis showing representative cleavages and secretions of caspase-1, IL-1β, and IL-18 in the infected BMDMs at 20 h after infection. BMDMs were isolated from WT, *Nlrp3*^−/*−*^, *Nlrp6*^−/*−*^, *Pycard*^−/−^, *Aim2*^−/*−*^ or *Casp11*^−/*−*^ mice. (B) TNF-α production, (C) IL-1β production, (D) IL-18 production, as determined by ELISA; representative of at least 3 experiments. The error bars represent the means ± SD. *P < 0.05, **P < 0.01; one-way ANOVA for (D).

We next investigated which components are necessary for *M*. *ulcerans* infection to activate the inflammasome using BMDMs derived from gene-knockout mice. The results of Western blot analysis showed that wild-type (ShT-P) or plasmid-cured (ShT-N) *M*. *ulcerans* still activated caspase-1, but only reduced amounts of the cleaved form of IL-1β and IL-18 were found in the supernatants of NLRP3-deficient cells, suggesting a partial dependency on NLRP3 (Fig 3A). In contrast, caspase-1 activation and cleavage/secretion of IL-1β or IL-18 in BMDMs infected by the *M*. *bovis* BCG strain occurred in an NLRP3-dependent manner. Use of adaptor protein ASC-deficient (*Pycard*^-/-^) BMDMs definitively showed that ASC is essential for both the caspase-1 activation and cleavage/secretion of the cytokines that were induced by all the bacterial strains. We also observed no suppressive effect on NLRP6 or AIM2, or caspase-11-deficient cells. Activation of the inflammatory responses in the cells infected with *M*. *ulcerans* was not mediated by non-canonical pathways involving caspase-11. Interestingly, inflammasome activation by *M*. *bovis* BCG occurred in a caspase-11-dependent manner. These data suggest that the induction of inflammasome activation by *M*. *ulcerans* requires Nod-like receptors other than NLRP6 or AIM2, in addition to NLRP3.

We examined the amounts of the cytokines secreted into the supernatants by ELISA. Secretion of TNF-α from cells infected with the BCG or plasmid-cured strain (ShT-N) was induced in a manner that was independent of both NLRP3 and ASC (Fig 3B). Consistent with the results of Western blotting, markedly reduced amounts of IL-1β were secreted from infected NLRP3-deficient macrophages, and the secretion of IL-1β was completely suppressed from infected ASC-deficient BMDMs (Fig 3C). On the other hand, the results in regard to inhibition of IL-18 secretion were unclear, because of the limited detection sensitivity in the lower concentration range of the ELISA system. However, the secretion of IL-18 induced by infection with all the strains was significantly suppressed in the NLRP3- or ASC-deficient macrophages (Fig 3D).

Inflammasome activation and secretion of IL-1β/IL-18 following infection with *M*. *ulcerans* strain Agy99, *M*. *marinum*, or *M*. *pseudoshottsii* were also completely abolished in ASC-deficient macrophages (S2 Fig). Dependency on NLRP3 was observed in the *M*. *pseudoshottsii*-infected cells, but only partially in the *M*. *marinum*-infected cells.

### IL-18 attenuates the progression of skin ulceration by *M*. *ulcerans* infection

**Fig 4:**
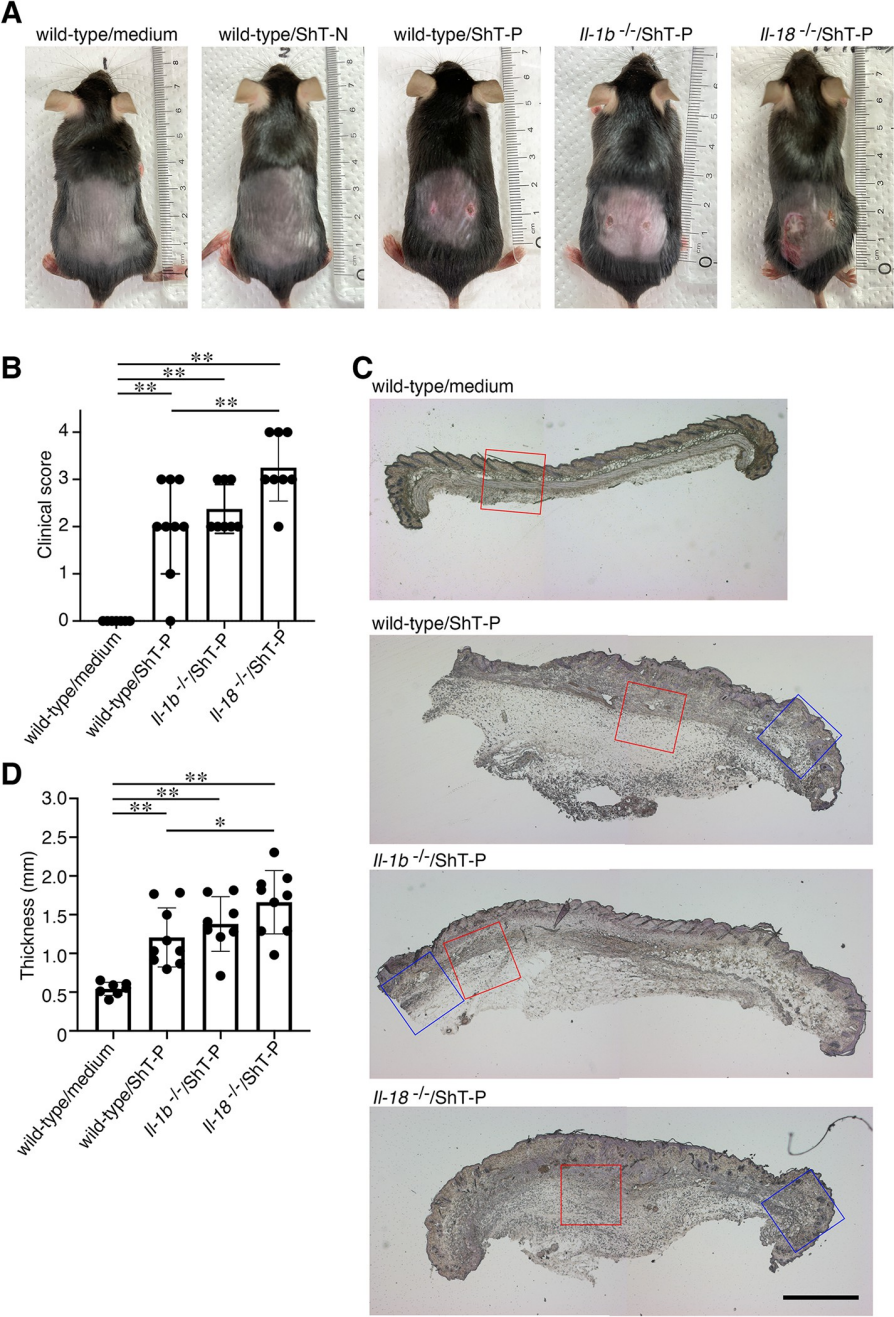
IL-18 attenuates the progression of skin ulcerations following infection with *M*. *ulcerans*. (A) Representative skin lesions on Day 14 in WT, *Il1b*^−/*−*^, and *Il18*^−/*−*^ mice infected with *M*. *ulcerans* strains. (B) Severity of the skin lesions in the infected mice. (C) Representative hematoxylin and eosin (H&E)-stained sections of skin tissue prepared from animals infected with a wild-type *M*. *ulcerans* strain. The red rectangles indicate lipoarabinomannan (LAM)-positive regions, and the blue rectangles indicate the surrounding regions (Fig 5A). Bar, 1 mm. (D) Skin thickness of the infected mice on day 14. The error bars represent the means ± SD. *P < 0.05, **P < 0.01; (B) and (D) n = 7–9, one-way ANOVA.

Since IL-18 is exclusively secreted from macrophages infected with wild-type *M*. *ulcerans*, we next examined the role of this cytokine on the progression of BU. First, we established a subcutaneous infection model using C57BL/6 mice. The wild-type *M*. *ulcerans* ShT-P strain was administered s subcutaneously into the shaved dorsal skin of the mice; a negative group (Middlebrook 7H9 media only) and a group infected with the plasmid-cured ShT-N strain were also used as controls. At 14 days post-infection, the skin lesions were evaluated. Localized nodules were observed in the mice infected with the wild-type strain, but not in the negative control group (media only) or group infected with the plasmid-cured mycolactone-negative strain (Fig 4A). Then, we examined the functional roles of IL-18 in the disease progression using IL-18-deficient mice and IL-1β-deficient mice. We anticipated that the lesions in the IL-18-deficient mice would be attenuated, since we speculated that IL-18 might accelerate skin inflammation through a mechanism phenotypically similar to that involved in the effect of anti-IL-18 therapy in autoinflammatory IL-18-driven diseases, such as familial Mediterranean fever and systemic juvenile idiopathic arthritis [27–29]. Unexpectedly, however, we found that the skin lesions in the IL-18-deficient mice infected with the wild-type strain were, in fact, exacerbated, with extensive ulcerations visible on the skin of the animals (Fig 4A). In contrast, the localized nodules on the skin observed in the infected IL-1β-deficient mice were comparable to those observed in the wild-type mice, suggesting that IL-18, but not IL-1β, exerts a protective role against disease progression. The exacerbation of the skin lesions in the infected IL-18-deficient mice was confirmed by the higher clinical score in these mice, as compared with the scores in the other groups (Fig 4B). Although hematoxylin and eosin (H&E)-stained sections of infected skin revealed hyperplasia of the dermis and subcutaneous tissue in all of the infected wild-type, IL-1β-deficient, and IL-18-deficient mice (Fig 4C), the thicknesses of these tissues in the IL-18-deficient mice were significantly greater than those in the other groups (Fig 4D).

### IL-18 controls the accumulation of neutrophils in the skin lesions, whereas IL-1β is involved in eradicating the infecting mycobacteria

**Fig 5:**
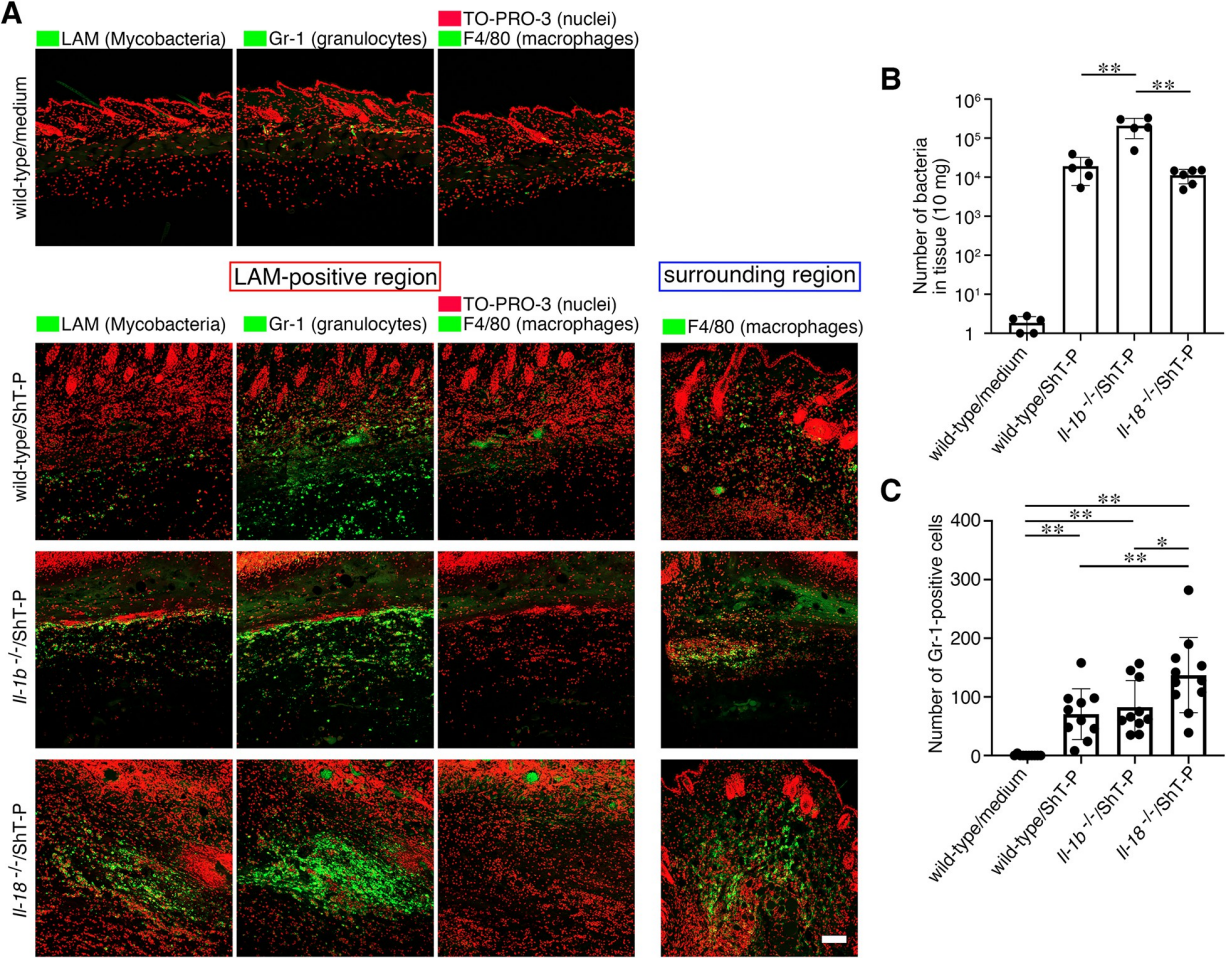
IL-18 controls the accumulation of neutrophils in the infected lesions, whereas IL-1β is involved in eradicating the infecting mycobacteria. (A) Representative images following immunostaining with anti-LAM (Mycobacteria), anti-Gr-1 (granulocytes), and anti-F4/80 (macrophages) of the LAM-positive region or surrounding region. The nuclei were counterstained with TO-PRO-3. Bar, 100 μm. (B) Quantification of the number of bacteria in the infected tissue. (C) Quantification of the number of Gr-1-positive cells (granulocytes) in the infected areas under magnification (x20). The error bars represent the means ± SD. *P < 0.05, **P < 0.01; (B) n = 5–6, (C) n = 10–12, one-way ANOVA.

We examined the bacterial colonization of the skin tissue by immunohistochemistry using mycobacteria-specific anti-lipoarabinomannan (LAM) antibody. The results revealed diffuse localization of mycobacteria in the subcutaneous tissue of the wild-type, IL-1β-deficient, as well as IL-18-deficient mice, but not in the uninfected control mice (Fig 5A). The number of bacteria in the infected skin tissue was then quantified using real-time PCR. Interestingly, the number of mycobacteria in the infected IL-1β-deficient mice was tenfold higher than that in skin tissue specimens obtained from the other infected mouse groups (Fig 5B). These results suggest that even though mycolactone secreted by *M*. *ulcerans* suppresses IL-1β production in the infected macrophages at the cellular level, IL-1β is somehow functional and involved in the inhibition of bacterial growth or eradication at the tissue level. The localization of granulocytes, including neutrophils and macrophages, in the skin tissue regions colonized by the mycobacteria was examined by immunohistochemistry using the Gr-1 and F4/80 antibody for neutrophils and macrophages, respectively. Neutrophil accumulation in the areas colonized by the bacteria was observed in the wild-type, IL-1β-deficient, as well as IL-18-deficient mice (Fig 5A). Quantification of the number of neutrophils accumulated in the infected skin areas revealed a significantly higher number in the IL-18-deficient mice as compared with that in the other infected mouse groups (Fig 5C). On the other hand, neutrophils were nearly absent in the skin of the uninfected control mice.

As compared with the number of macrophages, the number of migrated neutrophils in the skin was not affected by the activity of mycolactone. However, numerous macrophages were observed in the region (blue rectangles in Fig 4C) surrounding the infected areas of the skin (red rectangle in Fig 4C). These results suggest that *M*. *ulcerans* can eliminate macrophages, but not neutrophils, via mycolactone-mediated cytotoxicity. Taken together, our results suggest that IL-18 controls the migration of neutrophils and the inflammation induced by these cells, while IL-1β plays a role in eradicating the causative *M*. *ulcerans* bacteria from the infected subcutaneous lesions.

## Discussion

Mycolactone produced by *M*. *ulcerans* is the only reported virulence factor until date in Buruli ulcer. Functionally, mycolactone was at first thought to only suppress protein synthesis and secretion. On the other hand, recent reports have also shown mycolactone-induced inflammasome activation in the macrophages, followed by IL-1β secretion [21,22]. To further investigate these conflicting actions of mycolactone, we investigated cytokine induction and inflammasome activation in infected murine macrophages using an in vitro model of infection with viable wild-type *M*. *ulcerans* and mycolactone-deficient plasmid-cured strains. Cytokine induction, including that of IL-1β, was clearly suppressed in a mycolactone-dependent manner. On the other hand, the infection triggered activation of caspase-1 in a mycolactone-independent manner. Remarkably, constitutively produced proIL-18 was cleaved and mature IL-18 was, in fact, actually released from the macrophages infected with wild-type *M*. *ulcerans*. Inflammasome activation by *M*. *ulcerans* is dependent on adaptor protein ASC, but only partially dependent on NLRP3. The functional role of IL-18 in disease progression was examined using a subcutaneous infection model and IL-18-deficient mice. We found that IL-18 negatively regulated the severity of the skin inflammation induced by the bacterial infection, whereas IL-1β was involved in controlling the bacterial burden. Based on our findings, we propose that IL-18, which is induced in a mycolactone-independent manner, plays a key role in the regulation of inflammation in BU.

We also found mycolactone-dependent suppression of proIL-1β expression in the infected macrophages. The results observed in the cells infected at a low MOI, which does not induce rapid LDH release, lent support to this finding. At least, the suppressive effect was not attributable to cell death of the infected macrophages. Mycolactone suppresses the proIL-1β at the transcriptional level, suggesting that mycolactone might inhibit IL-1β expression via a mechanism independent of the Sec61-mediated secretion system. Previous studies have shown that mycolactone induces IL-1β secretion [21,22]. We speculate that the suppression of cytokines by *M*. *ulcerans* infection might differ depending on the *M*. *ulcerans* strain used to infect the cells or the type of mycolactone produced by the strains. Further studies are needed to understand the precise mechanisms of actions of mycolactone.

Recent reports have shown that IL-1β secretion from LPS-primed macrophages is induced by both mycolactone treatment and infection with *M*. *ulcerans* [21,22]. It should be noted that in our experiments, the analysis of the inflammatory responses in the macrophages following bacterial infection was performed without pre-treatment of the cells with LPS. If proIL-1β expression is induced by LPS prior to bacterial infection or treatment with mycolactone, the resulting proIL-1β could be cleaved by activated caspase-1 and mature IL-1β could be readily released, along with the induction of necrotic cell death by mycolactone. Recent report has shown that the release of IL-1β is induced by *M*. *ulcerans* infection and treatment with microvesicles containing mycolactone even in the absence of LPS [21]. The expression of proIL-1β could be activated by mycobacterial pathogen-associated molecular patterns which trigger Toll-like receptors (TLRs) such as TLR2. Inflammasome activation by *M*. *ulcerans* was completely inhibited in adaptor protein ASC-deficient macrophages, but residual partial activation still occurred in NLRP3-deficient cells. Possibly, another NLR protein might be involved in inflammasome activation by *M*. *ulcerans*. Foulon et al. also reported demonstrated NLRP3-dependent inflammasome activation by mycolactone [21]. However, we wish to discuss a little further about the K^+^ efflux induced by the cytotoxicity of mycolactone [17]. K^+^ efflux from the cytosol has been identified as a common switching signal for triggering NLRP3 [30]. Therefore, some level of lytic cell death with membrane integrity likely results in secondary engagement of the NLRP3 inflammasome pathway. However, since even low doses of mycolactone induced IL-1β release from the infected macrophages in the absence of any obvious cytotoxicity [21], it is possible that mycolactone can trigger the NLRP3 inflammasome via a pathway different from the K^+^ efflux-mediated pathway. In our study, inflammasome activation by the infected bacteria was closely associated with the release of LDH, suggesting that K^+^ efflux plays a major role in the activation. However, the possibility of differences in effects among mycolactone types produced by *M*. *ulcerans* strains cannot be ruled out.

Expression of IL-18 was first identified in Kupffer cells and macrophages [31], and also epidermal keratinocytes [32]. IL-18 is constitutively present in its precursor form in blood monocytes, macrophages, and dendritic cells [28]. In our experimental mouse model of subcutaneous infection, the supposed cell lineage may include macrophages or Langerhans cells. In general, IL-18 is known as a strong inducer of IFN-γ production by activated T cells. Several studies have shown suppression of IFN-γ production in patients of BU [33,34]. Studies have also demonstrated a Th2 response in BU patients [35] and an inverse correlation between the expression level of IFN-γ in the skin macrophages and the severity of BU [36]. IFN-γ-deficient mice exhibited more rapid progression of the skin ulcerations in BU [37]. Furthermore, the susceptibility to BU has been demonstrated to be associated with *IFNG* gene polymorphisms [38]. These findings suggest a strong association between suppression of IFN-γ-mediated immune responses and the rate of disease progression in BU. Our observation of the exacerbation of skin lesions in the IL-18-decient mice could be related to further suppression of the IFN-γ-mediated host response.

On the other hand, while IL-18 is known to enhance Th2 differentiation [39], it can also repress the Th2 response in vivo. IL-18-deficient mice show enhanced allergen-induced inflammation and exacerbation of chronic helminthic infections [40,41], suggesting the context-dependent role of IL-18 in type 2 immunity. Considering the roles of IL-18 in BU, it is possible that IL-18 may protect against disease progression by modifying the Th2 response.

IL-18 was also demonstrated to protect against colitis in a previously reported mouse model [42–44]. In contrast, inhibition of IL-18 has also been shown to exert protective effect in experimental colitis models [45–48]. These conflicting findings have led to much controversy and discussion, and the roles of IL-18 in intestinal homeostasis and in the development of inflammatory diseases of the bowel are still uncertain. In the case of BU, although we have suggested a protective role of IL-18, further investigation is necessary to clarify the roles of IL-18 in the course of progression of the disease.

IL-1β is an important proinflammatory cytokine. While IL-1β is mainly induced in macrophages during inflammation, its induction in neutrophils has also been demonstrated in several examples of bacterial infection [49–51]. We demonstrated that IL-1β induction was inhibited in macrophages infected with wild-type *M*. *ulcerans* in vitro. In our skin infection model in mice, the resident macrophages disappeared in areas colonized by the bacteria. We speculate that the migrated neutrophils might also produce IL-1β, which is involved in bacterial eradication. Alternatively, macrophages localized at a distance from the areas colonized by the mycobacteria may produce IL-1β. In fact, IL-1β-producing cells were observed at some distance from areas colonized by the acid-fast bacteria in biopsy specimens obtained from patients with BU [21]. Such cells might be activated with induction of IL-1β in response to low concentrations of mycolactone that have diffused farther from the areas of bacterial colonization [52], while the bacterial colonization might be controlled by activated neutrophils.

Understanding the molecular mechanisms by which skin inflammation is triggered in BU is essential for identifying the factors involved in disease progression, as well as preventing the prognostic symptoms and complications. Indeed, we observed many patients with sequelae, such as bony deformities, in endemic areas of Ghana. Antibiotic treatment alone is not sufficient for the treatment of Buruli ulcer. Our results shed light on the etiology of Buruli ulcer and provide insight into the relationships between inflammasome activation and cytokine functions.

## Materials and methods

### Ethics statement and mice

All the animal experiments conducted in this study were performed in accordance with the Guidelines for Animal Experimentation of the Japanese Association for Laboratory Animal Science. The Institutional Animal Care and Use Committee of Tokyo Medical and Dental University approved all the protocols prior to use (approval number: A2021-118A). The experimental protocols involving the use of a Living Modified Organism and gene-knockout mice were approved by the Genetically Modified Organisms Safety Committee of Tokyo Medical and Dental University (approval number: G2018-021C10). C57BL/6 mice were purchased as the WT mice from Japan SLC (Tokyo, Japan). NLRP3-deficient (*Nlrp3*^−/*−*^) [53], NLRP6-deficient (*Nlrp6*^−/*−*^) (generated in our laboratory), ASC-deficient (*Asc*^−/−^ or *Pycard*^−/−^) [54], caspase-11-deficient (*Casp11*^−/*−*^) (provided by Dr. Masahiro Yamamoto), IL-1β-deficient (*Il1b*^−/*−*^) [55], and IL-18-deficient (*Il18*^−/*−*^) [56] mice with a C57BL/6 background were housed in a specific-pathogen-free facility. The bone marrow of AIM2-deficient (*Aim2*^−/*−*^) mice was provided by Dr. Yasushi Kawaguchi.

### Bacterial strains

*M*. *ulcerans* subsp. *shinshuense* wild-type ShT-P and the isogenic plasmid-cured strain ShT-N [57], *M*. *ulcerans* Agy99 (provided by Dr. Mineo Watanabe) and *M*. *bovis* BCG strains were used in this study. *M*. *marinum* JCM17638 and *M*. *pseudoshottsii* JCM15466 were provided by the Japan Collection of Microorganisms, RIKEN BRC, which is a participant of the National BioResource Project of MEXT, Japan.

The handling of the *Mycobacterium* strains under biosafety level 2 conditions was approved by the Safety Control Committee for Pathogenic Microbes of Tokyo Medical and Dental University (approval number: M22019-004c3). Bacteria were cultured at room temperature (37°C for BCG strain) in Middlebrook 7H10 agar medium (Becton Dickinson) supplemented with 0.5% glycerol and 10% OADC Enrichment (Becton Dickinson). Mycobacteria grown on a solid plate were resuspended in 7H9 middlebrook medium and the optical densities (ODs) were measured at 540 nm. The approximate bacterial count was calculated as 1OD ~1.0 x 10^8^/ml, based on counting of the CFUs on the plates.

### Cells and infection

Bone marrow-derived macrophages (BMDMs) were prepared from the femurs of the above-mentioned experimental mice and cultured for 7 days in RPMI 1640 (Sigma) containing 10% FBS and 1% penicillin/streptomycin, supplemented with 30% supernatant of mouse L929-cell producing G-CSF. The cells were then infected with *M*. *ulcerans* strains at a MOI of 40 or treated with an equivalent volume of the corresponding Middlebrook 7H9 medium (Becton Dickinson).

### Western blotting

BMDMs were seeded at 1.6 x 10^6^ cells/well in 6-well plates containing RPMI 1640 supplemented with 10% FBS. Post-infection, the cells were lysed with lysis buffer (25 mM Tris-HCl, pH7.4, 150 mM NaCl, 1%NP-40, complete protease inhibitor cocktail; Roche Diagnostics), and the supernatants were precipitated by the addition of 6% trichloroacetic acid. The samples were loaded onto 15% SDS-PAGE. The following antibodies were obtained commercially; mouse anti-caspase-1 (p20) (Casper-1; Adipogen, AG-20B-0042), goat anti-mouse IL-1β (R&D, AF-401-NA), rabbit anti-mouse IL-18 (BioVision, 5180R-100), and anti-actin (Merck, MAB1501) antibody.

### ELISA and cytotoxicity assay

BMDMs were seeded at a density of 4 x 10^5^ cells/well in 24-well plates containing RPMI 1640 supplemented with 10% FBS. At the times indicated after the infection, the cytokines released into the culture supernatants were quantified using ELISA kits. The following ELISA kits were purchased commercially; mouse IL-1β (Invitrogen, 88-7013-88), mouse IL-18 (Invitrogen, BMS618-3), and mouse TNF-α (Invitrogen, 88-7324-88) kits. The lactate dehydrogenase (LDH) activity in culture supernatants of the infected cells was measured using a CytoTox 96 assay kit (Promega), in accordance with the manufacturer’s protocol.

### Subcutaneous infection of mice

Twenty-five microliters of a bacterial suspension containing 2.5x10^5^ CFU of *M*. *ulcerans* in Middlebrook 7H9 broth were injected subcutaneously into the shaved dorsal skin of six-week-old female C57BL/6 mice. Uninfected control mice received the same volume of just the 7H9 broth. At 14 days post-infection, the mice were euthanized, and their skin lesions were examined. The clinical severity score of the lesions was determined using a 5-point scale: 0, no symptoms; 1, localized swelling; 2, localized nodule with induration; 3, localized ulcer; and 4, diffuse ulcer with bleeding (by reference to the WHO fact sheets for Buruli ulcer: https://www.who.int/news-room/fact-sheets/detail/buruli-ulcer-(mycobacterium-ulcerans-infection)).

### Histochemical analysis

Extracted skin lesions were fixed in 4% paraformaldehyde for 24 h at 4°C; the tissues were then immersed overnight in 10% sucrose, 30% sucrose, or OCT compound, followed by embedding in OCT compound. Seven-micrometer-thick sections were prepared and stained with hematoxylin and eosin (H&E), in accordance with the appropriate standard protocol. For immunofluorescence staining, the tissue slices were permeabilized and blocked in 25 mM Tris-HCl, pH7.4, 150 mM NaCl, 0.2% saponin, 10% blocking One (Nacalai Tesque). The mycobacteria were stained with anti-Mycobacteria LAM (ViroStat, 5179) after treatment with fluorochrome-conjugated secondary antibody. To visualize neutrophils and macrophages, the following antibodies were obtained from commercial sources; anti-mouse Ly-6G/Ly-6C (Gr-1) (BioLegend, 108403) and anti-mouse F4/80 (BD, 565409), respectively. The samples were incubated with an amplifying secondary antibody (Simple stain mouse MAX-PO, Nichirei, 414311) after reacting with the primary antibody, then stained using a tyramide-based technique (TSA Fluorescein, PerkinElmer). Images were obtained on the LSM 800 Airyscan confocal microscope (Carl Zeiss). The cell numbers in two randomly selected tissue areas (magnification, x20) were calculated using the ImageJ software.

### Quantitative PCR

Total RNA of the infected macrophages was isolated using the miRNeasy Mini kit (QIAGEN, 217004). Quantitative RT-PCR was performed using RNA-direct SYBR Green Realtime PCR Master Mix (TOYOBO) and the CFX96 Real-Time system (Bio-Rad). Quantitative comparison of the gene expression levels of mouse *Il1b* and *Tnfa* was performed by the ΔΔCT method, using the expression level of *Gapdh* as the reference gene. The primer sequences used for *Il1b*, *Tnfa* and *Gapdh* were in accordance with Origene. The High Pure PCR Template Preparation Kit (Roche) was used to extract DNA from the mouse skin samples. Quantitative PCR was performed using Thunderbird SYBR qPCR Mix (TOYOBO) and the CFX96 Real-Time system. To generate a standard curve for real-time PCR, DNA was extracted from a bacterial suspension containing 1 x 10^7^ cells and serial dilutions were prepared. One microliter of DNA solution corresponded to a defined number of CFU (1.6 x 10^1^ to 1 x 10^4^). The total numbers of bacterial cells in the skin samples were extrapolated from the averaged standard curve in accordance with the manufacturer’s instructions. The primer sequences for IS*2404* of *M*. *ulcerans* were F- GCGCAGATCAACTTCGCGGT and R- GCCCGATTGGTGCTCGGTCA.

### Quantification and statistical analysis

Results are shown as the means ± SD. Comparisons and statistical tests were performed as indicated in each figure legend. For comparisons of two groups, two-tailed unpaired *t*-tests were used. For comparisons of multiple groups, one-way ANOVA was used. The statistical analyses were performed using the GraphPad Prism 8 software. The *P* values denoted throughout the manuscript highlight biologically relevant comparisons. A *P* value of less than 0.05 was considered as indicative of significance, denoted as *P < 0.05, **P <0.01 for all analyses.

## Supporting information

S1 Fig Different behaviors of production of cytokines from BMDMs infected with Mycobacterium strains. **Related to Fig 1.** (A) TNF-α production, (B) IL-1β production, (D) IL-18 production, as determined by ELISA, of bone marrow-derived macrophages (BMDMs) supernatants at 6 or 20 h after infection with *Mycobacterium* strains. (C) Representative cleavage and secretion of caspase-1, IL-1β, and IL-18 by Western blot analysis. (E) Release of LDH from infected macrophages; representative at least 3 experiments. The error bars represent the means ± SD.
(TIF)

S2 Fig *Mycobacterium* strains trigger inflammasome activation in dependent on ASC but not NLRP3, NLRP6. **Related to Fig 3.** (A) Representative cleavage and secretion of caspase-1, IL-1β, and IL-18 of infected BMDMs by Western blot analysis. BMDMs were isolated from WT, *Nlrp3*^−/−^, *Nlrp6*^−/−^, *Pycard*^−/−^ mice. (B) TNF-α production, (C) IL-1β production, (D) IL-18 production, as determined by ELISA; representative at least 3 experiments. The error bars represent the means ± SD.
(TIF)

S1 Data Legends of S1 Data for graphs. Excel spreadsheet containing, in separate sheets, the underlying numerical data and statistical analysis for Figure panels 1A, S1A, 1B, S1B, 1D, S1D, 1E, S1E, 2B, 2C, 2D, 3B, S2B, 3C, S2C, 3D, S2D, 4B, 4D, 5B and 5C.
(XLSX)
