# Supplementary figures and images for: Inflammasome-triggered IL-18 controls skin inflammation in the progression of Buruli ulcer
Source: PLoS Pathog. 2023 Nov 1;19(11):e1011747. doi: 10.1371/journal.ppat.1011747 (PMC10619818; doi:10.1371/journal.ppat.1011747)

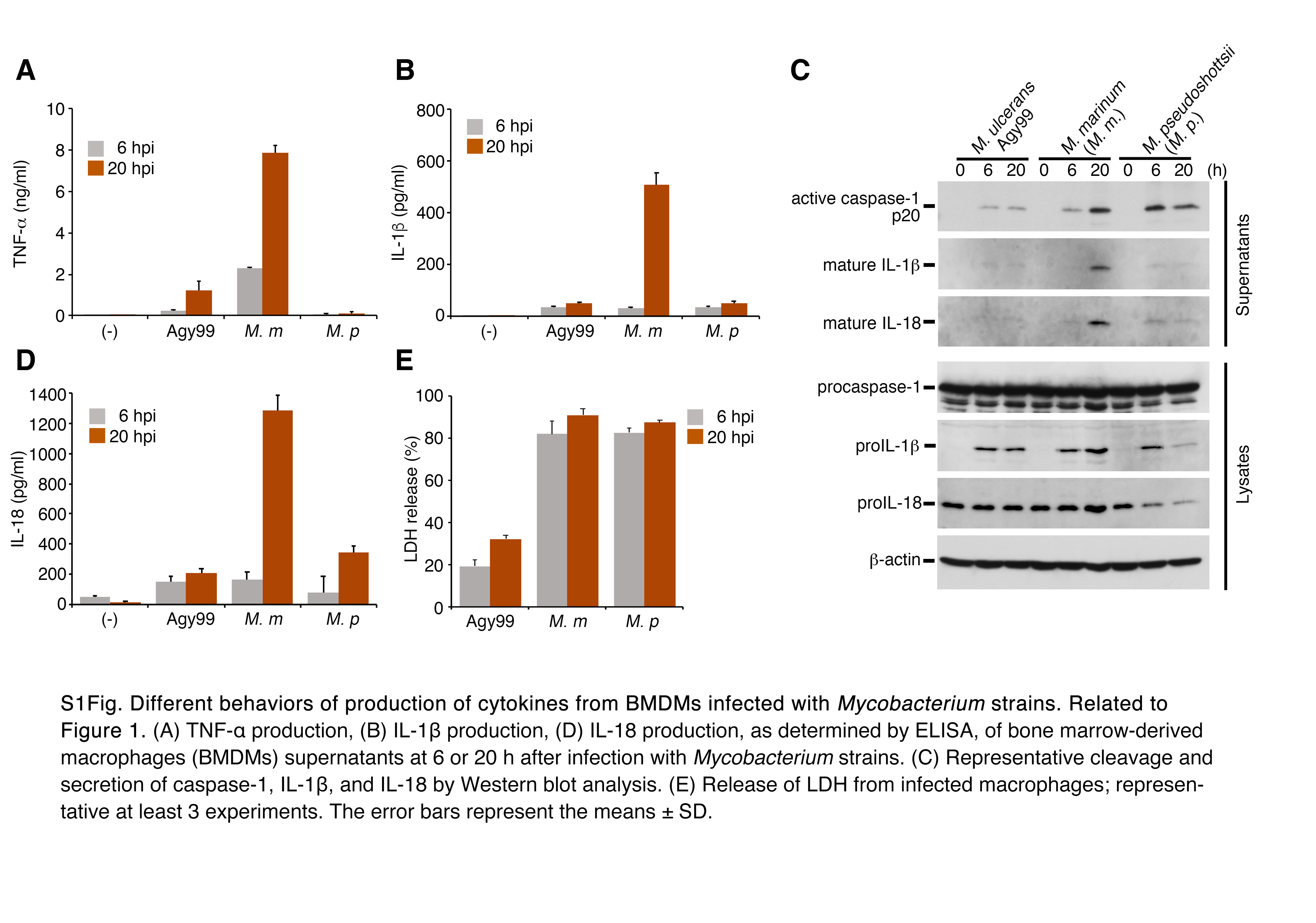

Supplement: S1 Fig — Related to Fig 1. (A) TNF-α production, (B) IL-1β production, (D) IL-18 production, as determined by ELISA, of bone marrow-derived macrophages (BMDMs) supernatants at 6 or 20 h after infection with Mycobacterium strains. (C) Representative cleavage and secretion of caspase-1, IL-1β, and IL-18 by Western blot analysis. (E) Release of LDH from infected macrophages; representative at least 3 experiments. The error bars represent the means ± SD. (TIF) [file ppat.1011747.s001.tif]

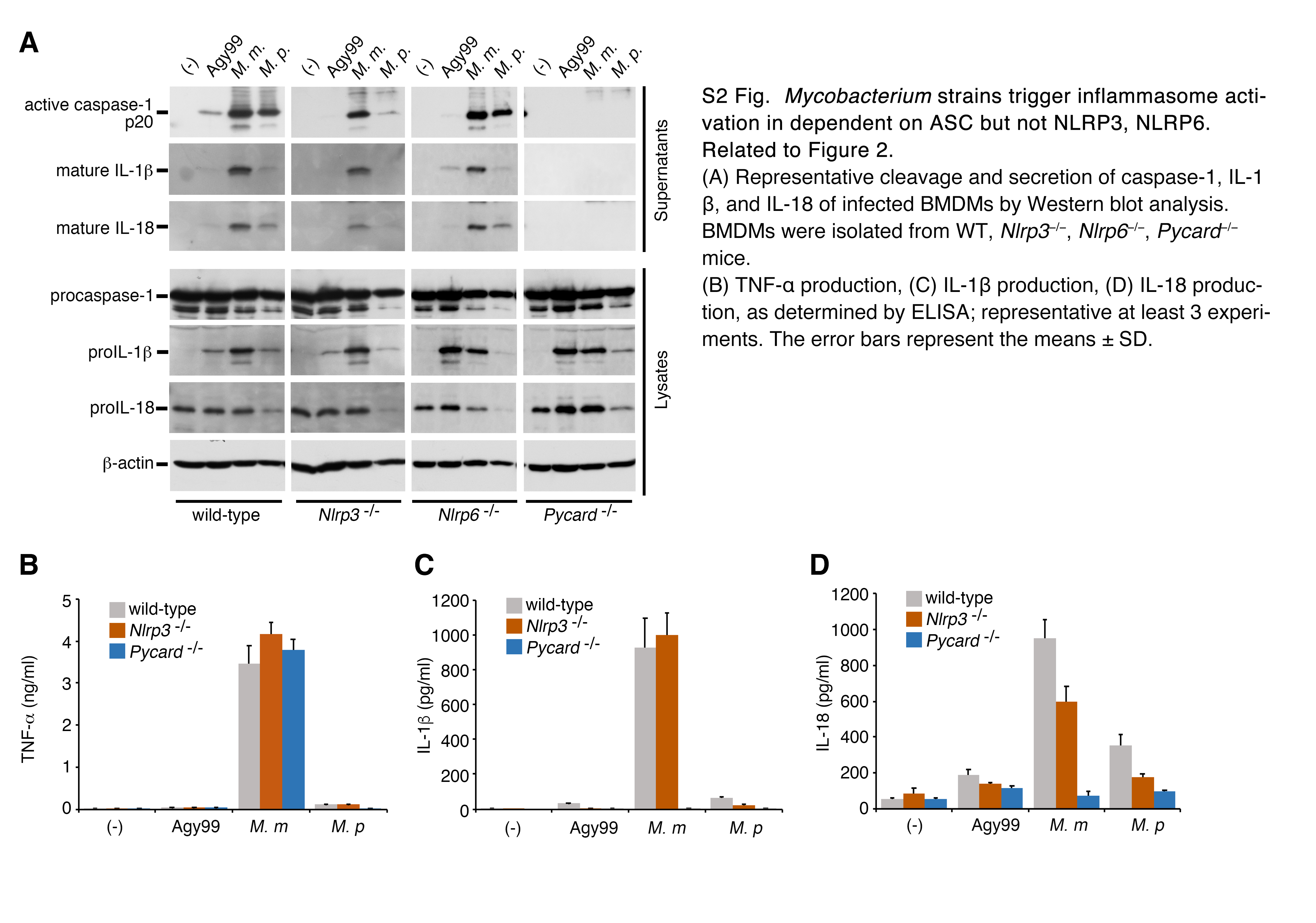

Supplement: S2 Fig — Related to Fig 3. (A) Representative cleavage and secretion of caspase-1, IL-1β, and IL-18 of infected BMDMs by Western blot analysis. BMDMs were isolated from WT, Nlrp3−/−, Nlrp6−/−, Pycard−/− mice. (B) TNF-α production, (C) IL-1β production, (D) IL-18 production, as determined by ELISA; representative at least 3 experiments. The error bars represent the means ± SD. (TIF) [file ppat.1011747.s002.tif]
